# Visualization of Allostery in P-Selectin Lectin Domain Using MD Simulations

**DOI:** 10.1371/journal.pone.0015417

**Published:** 2010-12-08

**Authors:** Shouqin Lü, Yan Zhang, Mian Long

**Affiliations:** 1 Key Laboratory of Microgravity, Institute of Mechanics, Chinese Academy of Sciences, Beijing, People's Republic of China; 2 National Microgravity Laboratory, Institute of Mechanics, Chinese Academy of Sciences, Beijing, People's Republic of China; 3 Center of Biomechanics and Bioengineering, Institute of Mechanics, Chinese Academy of Sciences, Beijing, People's Republic of China; University of South Florida College of Medicine, United States of America

## Abstract

Allostery of P-selectin lectin (Lec) domain followed by an epithelial growth factor (EGF)-like domain is essential for its biological functionality, but the underlying pathways have not been well understood. Here the molecular dynamics simulations were performed on the crystallized structures to visualize the dynamic conformational change for state 1 (*S*1) or state 2 (*S*2) Lec domain with respective bent (*B*) or extended (*E*) EGF orientation. Simulations illustrated that both *S*1 and *S*2 conformations were unable to switch from one to another directly. Instead, a novel *S*1' conformation was observed from *S*1 when crystallized *B*-*S*1 or reconstructed “*E*-*S*1” structure was employed, which was superposed well with that of equilibrated *S*1 Lec domain alone. It was also indicated that the corresponding allosteric pathway from *S*1 to *S*1' conformation started with the separation between residues Q30 and K67 and terminated with the release of residue N87 from residue C109. These results provided an insight into understanding the structural transition and the structure-function relationship of P-selectin allostery.

## Introduction

Selectin-ligand interactions play a crucial role in inflammatory and immune responses *via* mediating leukocyte tethering to and rolling on vascular surfaces. Selectin is characterized by an extracellular C-type lectin (Lec) domain, a single epidermal growth factor (EGF)-like domain, and a variable number of short consensus repeat (SCR) units homologous to complement regulatory proteins, a transmembrane region, and a cytoplasmic tail [1]. EGF domain is well-known to be functional in ligand recognition and cell adhesion [2], since Lec domain alone does support leukocyte adhesion but is insufficient for maximal binding whereas the concurrence of Lec and EGF (LE) domains constitutes the optimal recognition unit for leukocyte binding [3]. Presence of SCR domains enhances the leukocyte adhesion *via* presenting sufficient length and flexibility for a P-selectin molecule to bind to its counterpart ligand, P-selectin glycoprotein ligand 1 (PSGL-1) [4]. Particularly, an effective binding unit is formed by LE domains and SGP-3 peptide, a 19 N-terminal sulfoglycopeptide of PSGL-1 composed of three tyrosine sulfate residues Y605, Y607, and Y610, and a sLe^x^-modified glycan at T616 [5]. Despite the biological significance of LE domains is well defined [3], the structural bases of both Lec and EGF domains in regulating cell adhesion need to be further investigated.

**Figure 1:**
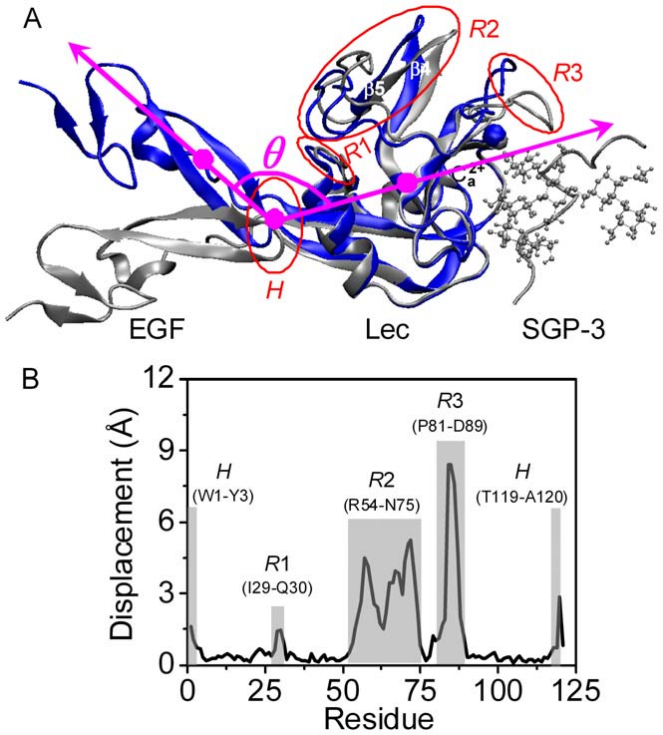
Conformational difference between unliganded and SGP-3 liganded P-LE domains. (*A*) Crystallized conformational difference between unliganded (*blue*) and SGP-3 liganded (*silver*) P-LE domains presented as *newcartoon*, calcium ion Ca^2+^ and SGP-3 glycan were presented as *VDW* and *CPK* respectively. Four distinct regions were illustrated as *red* for hinge (*H*) region, region 1 (*R*1), 2 (*R*2), and 3 (*R*3). Two structures were aligned upon alpha carbon atoms of Lec domain. The angle *θ* between two vectors (*magenta arrows*) connecting the geometric center of heavy atoms at *H* (residues A120 and S121) to that of Lec domain (residues W1 to T119) and to that of main EGF domain (residues C122 to T141) (*magenta points*) was also defined to measure the orientation of EGF to Lec domain. Anti-parallel β_4_ (I53-N56) and β_5_ (T59-W62) sheets of *R*2 were labeled for clarity. (*B*) Quantification of conformational difference of Lec domain (residues W1 to A120) between the two crystallized structures. The displacement was defined as the distance between heavy atom centers of each residue by aligning the alpha carbon atoms of Lec domain. Four regions presented in (*A*) were correspondingly highlighted as *grey stripes* with the displacement >1 Å, and the calcium ion was identified at the final residue 121.

Orientation and conformation of Lec and EGF domains are important to possess their functions. Comparison of crystallized structures of unliganded P-LE domains with liganded P-LE-SGP-3 or P-LE-sLe^X^ (sialic acid *X*) complex make it possible to elucidate the structure-function relationship of P-selectin-PSGL-1 interactions at atomic level [6]. For example, two distinct conformations of Lec domain with different orientations of EGF domain are visualized, one is so-called *state* 1 Lec domain (denoted as *S*1) followed by *bent* EGF domain (denoted as *B*) with a closed angle from EGF to Lec domain, and the other is *state* 2 Lec domain (denoted as *S*2) followed by *extended* EGF domain (denoted as *E*) with an open angle (*cf*. *blue and silver newcartoons* in Fig. 1*A*). The conformational difference between *S*1 and *S*2 Lec domain mainly lies in four specific regions (*cf*. *H*, *R*1, *R*2 and *R*3 highlighted as *red circles* in Fig. 1*A*). For example, *R*3 loop of *S*1 Lec domain (P81-D89) involved directly in ligand binding site parallels the binding interface and points away from the major binding sites while that of *S*2 Lec domain orients vertically outwards the binding interface and points toward those sites. Noting that *B*-*S*1 conformation appears in the unliganded P-LE or liganded P-LE with sLe^x^ and *E*-*S*2 conformation is found in the liganded P-LE-SGP-3 complex, it is evident that the conformation of Lec domain is associated with the orientation of EGF domain and that the allosteric pathways of conformational transition is expected. The structural analysis is also crucial to the binding capacity of a selectin molecule to PSGL-1 ligand under blood flow, since the conformational change of Lec domain is usually accompanied with the action of mechanical force [7].

Allostery is a common feature for biomolecular function observed in such the proteins as FimH [8], integrin [9], [10], von Willebrand Factor (vWF) [11], and myosin [12]. P-selectin allostery was observed *via* introducing a glycan wedge between Lec and EGF domains [13] or mutating A28H residue near the hinge of Lec and EGF interface [14], which in turn enhanced the binding affinity to PSGL-1 ligand. An allosteric model was proposed based on crystallized structures to predict the pathway of P-LE domains from a low-affinity (the same as *S*1) to the high-affinity conformation (the same as *S*2) [7] where the conformational transition started with the destroy of interaction network among those residues locating at the interface between Lec and EGF domains and was terminated by the redistribution of hydrogen bonds (Hbond) between “switch2” and “switch3” (corresponding to respective *R*2 and *R*3 regions in the current study; *cf.* Fig. 1*A*). It still remains unknown, however, whether the allostery from *S*1 to *S*2 takes place directly and what are the underlying structural bases. Here we explored the allosteric dynamics of P-selectin Lec domain at atomic level to understand the structural evolution of P-selectin allostery. Impacts of Lec domain stability, EGF domain orientation, and SGP-3 ligation on conformational change of Lec domain were tested using molecular dynamic (MD) simulations.

## Methods

The crystallized *B*-*S*1 structures of unliganded P-LE domains (PDB code: 1G1Q) and of sLe^X^-liganded E-LE domains (PDB code: 1G1T), and the *E*-*S*2 structure of SGP-3-liganded P-LE domains (PDB code: 1G1S) were employed as initial structures. Here a strontium ion Sr^2+^ was replaced by a calcium ion Ca^2+^ for the *E*-*S*2 structure. Every simulation system was built by solvating the target molecule into a rectangular water box and neutralized with ∼100 mM Na^+^ and Cl^−^ ions added to mimic the physiological ionic concentration, and was then equilibrated no less than 5 nanosecond (*ns*) using a NAMD program [15], a CHARMM22 all-atom force field for protein [16], and a self-built force field for six sugar residues and a tyrosine sulfate residue in SGP-3 ligand [17]. An integration time step of 1 femtosecond (*fs*) and the periodic boundary conditions were applied in the simulations. Prior to equilibration process, energy minimization was initiated with 10000 steps by fixing backbone atoms of protein or heavy atoms of sugar followed by additional 10000 steps with all atoms free. System heating was then performed from 0 to 300 *K* at 30 *K* increment every 5 picosecond (*ps*). A smooth (10–12 Å) cutoff and the Particle Mesh Ewald (PME) method were employed to calculate van der Waals forces and full electrostatics, respectively. The 300 *K* heat bath was manipulated under Langevin thermostat, and the 1 *atm* pressure was controlled by Nosé-Hoover Langevin piston method.

Steered molecular dynamics (SMD) simulations were also conducted in some cases to test the impact of external force on conformational stability of Lec domain during P-LE unfolding or P-LE-SGP-3 complex dissociation. Here *B*-*S*1 structure of P-LE domains was forced to unfold using *cf*-SMD algorithm with a constant force of 100 pN along the vector from the fixed C-terminal atom L116-C_α_ of Lec domain to the pulled C-terminal atom D158-C_α_ of EGF domain. P-LE-SGP-3 complexes with different conformations were forced to dissociate using *cv*-SMD algorithm where C-terminal atom P618-C_α_ of SGP-3 ligand peptide was pulled *via* a spring with a spring constant of 70 pN/Å at a constant speed of 0.01 Å/*ps* along the vector from fixed C-terminal atom D158-C_α_ of EGF domain to the pulled end. Every system was equilibrated no less than 2 *ns* before SMD simulations.

Three types of structural analyses were conducted to illustrate the conformational characteristics of molecule of interest. The first was to quantify the orientation from EGF to Lec domain using the angle *θ* between two vectors connecting respectively the geometric center of heavy atoms of Lec-EGF interface hinge (residues A120 and S121) to that of Lec domain (residues W1 to T119) and to that of main EGF domain (residues C122 to T141) (*magenta circles and lines* in Fig. 1*A*). The next was to figure out the conformational change of Lec domain using the root of mean standard deviation (RMSD) of entire Lec domain or of specific regions, or the geometric center displacement of a residue by aligning the target to reference Lec domain, as well as the distance between specific residues. The final was to determine the interactions between Lec domain and SGP-3 ligand or protein and water molecules or among different regions of P-LE domains using the number of hydrogen bond (Hbond) with a donor-acceptor distance <3.5 Å and a donor-hydrogen-acceptor angle <45°. The system built-up and data analyses were performed using VMD program [18].

## Results

### Comparison of crystallized *B-S*1 and *E-S*2 conformations

The *B-S*1 conformation of P-selectin in the absence of ligand or soaked with sLe^X^ presented four distinct regions of Lec domain from those of *E-S*2 conformation with SGP-3 ligand [6], that is, the hinge region (denoted as *H*) of Lec and EGF interface involving two terminals of Lec domain (W1-Y3 and T119-A120), the loop region (denoted as *R*1) right ahead alpha helix 2 (I29-Q30), the anti-parallel beta sheets β_4_ and β_5_ and followed loop R54-N75 (denoted as *R*2), and the loop P81-D89 involving in ligand binding site (denoted as *R*3) (*red circles* in Fig. 1*A*). Here we focused on the conformational changes of the four regions and denoted the other regions of Lec domain as the rigid regions. The difference between *S*1 and *S*2 conformations was further quantified using the residue displacement by aligning *S*1 to *S*2 conformation, which was found to be >1 Å in all four regions (*grey stripes* in Fig. 1*B*).

The aforementioned differences between *S*1 and *S*2 conformations may lie in the following line of reasoning: the first is attributed to the conformational difference of Lec domain, the second is owing to the presence of SGP-3 ligand since the *B-S*1 structure is unliganded or sLe^X^-liganded and the *E-S*2 structure is SGP-3-liganded, and the third is referred to the orientation difference of EGF domain. To test the possibilities, five sets of simulations over eighteen runs were done upon crystallized unliganded and SGP-3-liganded P-LE and sLe^X^-liganded E-LE structures (Table 1), as described below.

**Table 1:** Summary of simulation set-up.

Set	System	Procedure	Duration (*ns*)	Objective
**I**	*S*1	Free equilibration	16	Stability of Lec domain alone
	*S*2	Free equilibration	20	
**II**	*S*1-SGP-3	Free equilibration	5	Impact of SGP-3 ligation
	*S*2-SGP-3	Free equilibration	5	
**III**	*E*-*S*1	Constraint of extended EGF orientation	10/9 (2 runs)	Impact of EGF orientation
	*B*-*S*2	Constraint of bent EGF orientation	10	
**IV**	*E*-*S*1 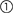	Constraint of *E*-EGF and residue W1 in *S*2 orientation	10	Allosteric pathway of Lec domain
	*E*-*S*1 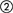	Constraint of *E*-EGF and residues W1, A28, and E34 in *S*2 orientation	10	
	*E*-*S*1 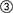	Constraint of *E*-EGF and residues W1, A28, I29, E34, and W62 in *S*2 orientation	10	
	*E*-*S*1 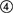	Constraint of *E*-EGF and residue D89 in *S*2 orientation	10	
	*B*-*S*2 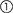	Constraint of *B*-EGF and residue W1 in *S*1 orientation	10	
	*B*-*S*2 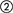	Constraint of *B*-EGF and residues W1, A28, and E34 in *S*1 orientation	10	
	*B*-*S*2 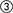	Constraint of *B*-EGF and residues W1, A28, I29, E34, and W62 in *S*1 orientation	10	
	*B*-*S*2 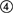	Constraint of *B*-EGF and residue D89 in *S*1 orientation	10	
**V**	*B*-*S*1	Free equilibration	45	Impact of EGF presence
	*B-S*1 (*E-LE*)	Free equilibration	45	
	*E*-*S*2	Free equilibration	30	

*S*1: state 1 Lec domain; *S*2: state 2 Lec domain; *B*: bent EGF orientation; *E*: extended EGF orientation. E-LE: E-selectin Lec and EGF domain.

### Impact of Lec domain alone

**Figure 2:**
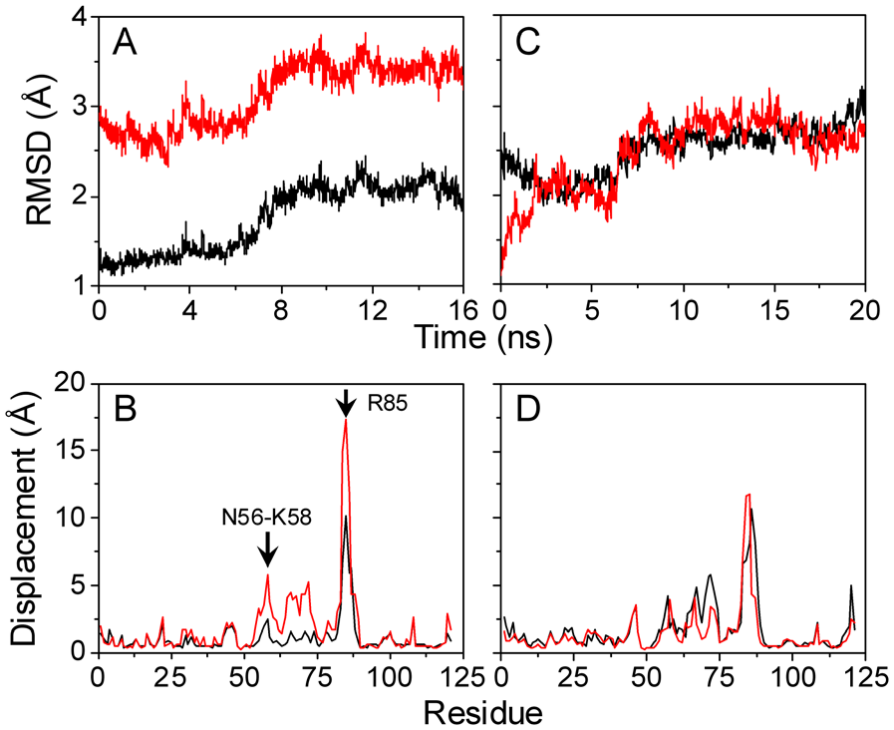
Stability of *S*1 or *S*2 Lec domain alone. Stability of *S*1 (*A*, *B*) or *S*2 (*C*, *D*) Lec domain alone was quantified by heavy atom RMSD evolution (*A*, *C*) and averaged displacement profile (*B*, *D*). Equilibrated *S*1 or *S*2 snapshots were aligned to crystallized *references S*1 (*black*) and *S*2 (*red*), respectively. RMSD for Lec domain and displacement for each residue were calculated upon alignment of alpha carbon atoms of Lec domain rigid regions after excluding the four regions of *H*, *R*1, *R*2, and *R*3. Averaged displacement from last 2-*ns* snapshots in each simulation was presented. N-terminal loop N56-K58 between anti-parallel β_4_ (I53-N56) and β_5_ (T59-W62) sheets of *R*2, and apex residue R85 of *R*3 were highlighted by arrows in (*B*).

*Set I* simulations were conducted to compare the conformational difference of Lec domain alone between the two structures (Table 1). Here EGF domain was deleted from crystallized *B-S*1 or *E-S*2 structure, and the two resulted structures (denoted as *reference S*1 and *S*2) were equilibrated for 16 and 20 *ns*, respectively (Fig. 2). To quantify the conformational changes, we first calculated the RMSD of Lec domain (Fig. 2*A, C*) and the displacement of heavy atom geometry center of each residue (Fig. 2*B, D*) *via* aligning the equilibrated snapshots to *reference S*1 (*black*) or *S*2 (*red*). As exemplified in Fig. 2*A*, the RMSD of equilibrated *S*1 structure exhibited a transition phase with ∼1.5 Å at <6 *ns* followed by ∼2.2 Å at >8 *ns* when aligning with *reference S*1 (*black*), while it yielded much high RMSD value ranging from ∼2.8 Å at <6 *ns* to ∼3.5 Å at >8 *ns* when aligning with alternative *reference S*2 (*red*). Averaged residue displacement calculated from last 2 *ns* equilibration presented the significant conformational change in the loop of *R*3 region, as exemplified by a maximal value of 10.1 and 17.4 Å for residue R85 (*arrow*), respectively (Fig. 2*B*). The slightly-high displacement was also found for the loop N56-K58 (*arrow*) between anti-parallel β_4_ and β_5_ sheets of *R*2 region. These data suggested that *S*1 Lec domain alone is not stable.

This was further tested by aligning the equilibrated *S*2 Lec domain to *reference S*1 or *S*2 for 20 *ns* simulation. Again, both RMSDs reached a high equilibrium value of ∼2.8 Å for last 12 *ns* (*black* and *red lines* in Fig. 2*C*) with the large displacement of the residues at the *R*3 loop and *R*2 (Fig. 2*D*), supporting the above observation. Taken together, these results indicated that either *S*1 or *S*2 Lec domain is an unstable structure with the instability of *R*3 for the former or of both *R*3 and *R*2 for the latter. The conformational interchange between *S*1 and *S*2 was not observed in the simulations.

### Impact of SGP-3 ligation

**Figure 3:**
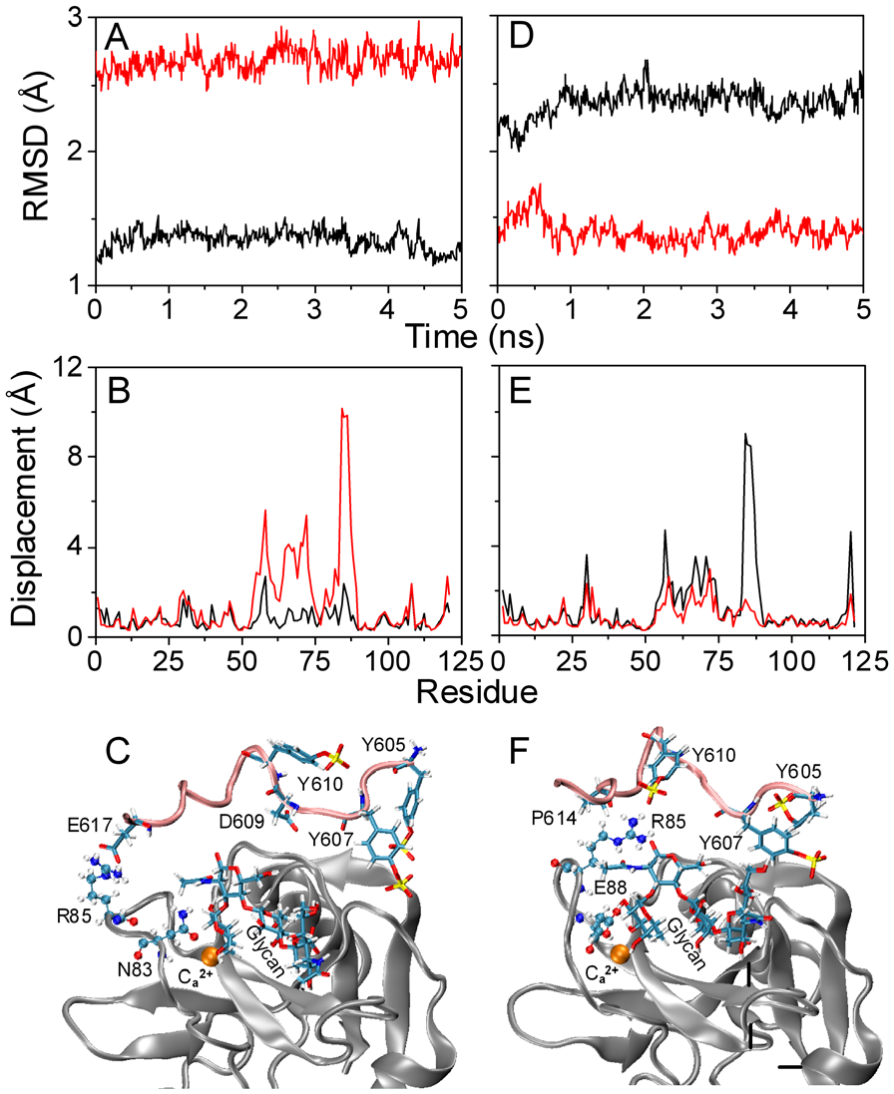
Impact of SGP-3 ligand on the stability of *S*1 or *S*2 Lec domain. Stability of *S*1 (*A*, *B*) or *S*2 (*C*, *D*) Lec domain interacting with SGP-3 ligand was quantified by RMSD evolution (*A*, *D*) and displacement profile (*B*, *E*) when aligning to crystallized *S*1 (*black*) and *S*2 (*red*) *references*. Calculations of RMSD and displacement were the same as those in Figure 2. Also illustrated were key interaction networks for *S*1 (*C*) and *S*2 (*F*) Lec domain with SGP-3 ligand where Lec domain and SGP-3 peptide were presented as *grey* and *pink newcartoon*, respectively. Key residues involved in Lec-SGP-3 interaction were presented as *named CPK* for Lec domain and *licorice* for SGP-3 peptide. Three sulfated tyrosines (Y605, Y607 and Y610) and glycan of SGP-3 ligand were demonstrated as *named licorice* and calcium ion was presented as *orange VDW*.

Next we tested the impact of presence of SGP-3 ligand on conformational difference of Lec domain. *Set II* simulations (Table 1) were done for 5 *ns* and the equilibrated conformation of *S*2-SGP-3 structure isolated from crystallized *E*-*S*2-SGP-3 complex was compared with that of *S*1-SGP-3 structure reconstructed *via* replacing *S*2 by *S*1 Lec domain. It was found that *S*1 Lec domain retained its original conformation with an equilibrated RMSD of ∼1.3 Å aligned to *reference S*1 (*black*) but high RMSD of ∼2.6 Å to *reference S*2 (*red*) (Fig. 3*A*). Combined with the observation of much smaller displacement for the former (the maximum of 2.4 Å; *black line*) than that for the latter (the maximum of 10.0 Å; *red*) (Fig. 3*B*), these data indicated that the presence of SGP-3 stabilized the conformation of *S*1 Lec domain (especially for *R*3 loop). Similarly, *S*2 Lec domain was also stabilized by SGP-3 ligation, as seen in an equilibrated RMSD of ∼2.4 Å and ∼1.3 Å to *reference S*1 (*black*) and *S*2 (*red*), respectively (Fig. 3*D*). Corresponding displacement profiles illustrated the same trend with small value to reference *S*2 (*red*) and large value to reference *S*1 (*black*) (Fig. 3*E*). Noting that the *R*3 is the most flexible region and that the residue R85 at the apex of *R*3 loop is the major binding site for SGP-3 ligand, our simulations demonstrated that a stable salt bridge between R85 and E617 of SGP-3 peptide was formed in *S*1-SGP3 complex (Fig. 3*C*) and that the interaction between R85 and sulfated tyrosine T610 of SGP-3 peptide was stably reserved in *S*2-SGP-3 complex (Fig. 3*F*). These results indicated that it is the binding of R85 with SGP-3 peptide that reduced the flexibility of *R*3 loop and stabilized its conformation. By contrast, the current simulations did not propose the allostery from *S*1 to *S*2 Lec domain when SGP-3 ligand was presented, suggesting that SGP-3 ligation is not directed to induce the allosteric transition of Lec domain. It was further noted that the structural stability induced by SGP-3 ligation was also observed when extending the simulations from 5 to 10 *ns* or even performing the 25-*ns* equilibrations for *B*-*S*1-SGP-3 and *E*-*S*2-SGP-3 complexes (*data not shown*), indicating these observations from the simulations were reliable.

### Impact of EGF orientation

**Figure 4:**
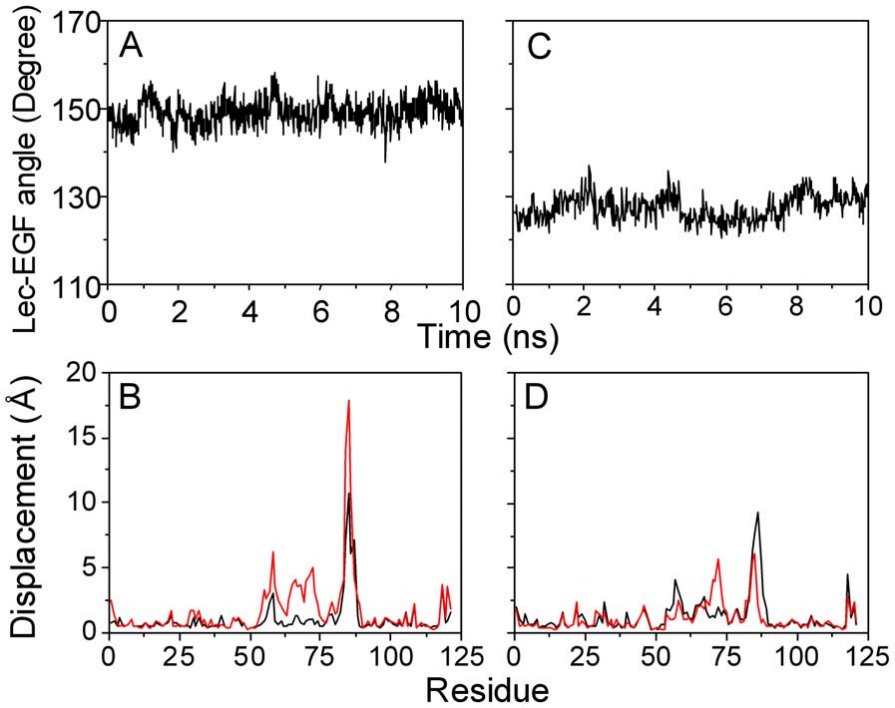
Impact of EGF orientation on the stability of *S*1 or *S*2 Lec domain. Stability of EGF orientation (*A*, *C*) or of *S*1 or *S*2 Lec domain conformation (*B*, *D*) with interchanged EGF orientation. EGF orientation, defined as the angle illustrated in Figure 1*A*, was quantified for *S*1 (*A*) or *S*2 (*C*) Lec domain. Displacement profiles, calculated as those in Figure 2, were measured for *S*1 (*B*) or for *S*2 (*D*) Lec domain upon aligning to crystallized *reference S*1 (*black*) and *S*2 (*red*) conformations.

EGF domain with residues S121-E158 distant from Lec-SGP-3 interface is necessary for optimal interaction between selectin and PSGL-1 ligand [3]. Previous reports of the distinct EGF orientations in crystallized *B*-*S*1 and *E*-*S*2 structures [6] and the conformational transition of Lec domain by the presence of EGF domain [7] implied the potential impact of EGF orientation on conformation of Lec domain. To test the hypothesis, two systems of “*E-S*1” and “*B-S*2” reconstructed by interchanging *B-*EGF domain with *E-*EGF domain between two P-LE structures were equilibrated for 10 *ns* with constrained backbone atoms of interchanged EGF domain (*Set III* in Table 1). As exemplified in Fig. 4, EGF orientation was enhanced to ∼149.0±2.9° for “*E-S*1” (Fig. 4*A*) but reduced to ∼127.3±3.0° for “*B-S*2” Lec domain (Fig. 4*C*). Conformational analysis indicated that *S*1 Lec domain was no longer stable with dramatic structural change of *R*3 (Fig. 4*B*) and that the conformational change of *S*2 Lec domain locating at *R*3 and the ending loop of *R*2 (*red*) was intermediate (Fig. 4*D*). These results indicated that the re-orientation of EGF domain induced the conformational change of Lec domain but not the direct interchange between *S*1 and *S*2 conformations, since the equilibrated conformation of *S*1 or *S*2 Lec domain obtained from “*E-S*1” or *“B-S*2” system was evidently different from that of crystallized *S*1 or *S*2 Lec domain (Fig. 4).

**Figure 5:**
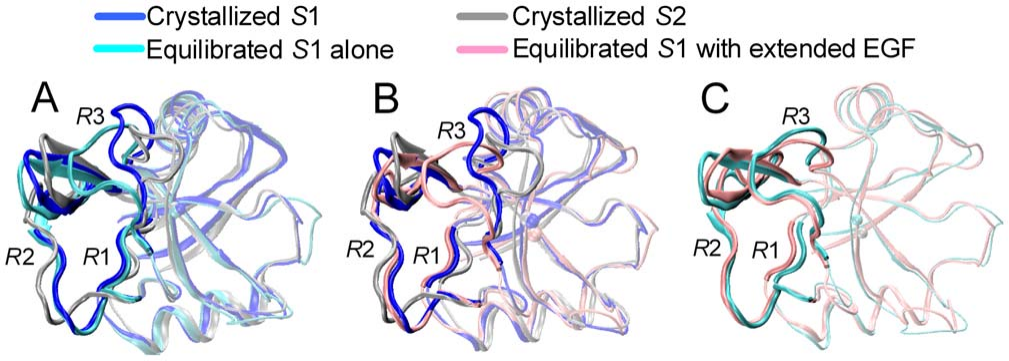
Conformational change of Lec domain. Equilibrated conformations of *S*1 Lec domain alone (*A*, *cyan*) or with the interchanged domain from *B* to *E*-EGF (*B, pink*) were superposed with the crystallized *S*1 (*blue*) and *S*2 (*silver*) *references*, respectively, by aligning the rigid regions of Lec domain. Conformational comparison between *A* and *B* was illustrated in *C*. *R*1, *R*2, and *R*3 were presented as *thick, opaque newcartoon* and the others were illustrated as *transparent newcartoon* for clarity.

**Figure 6:**
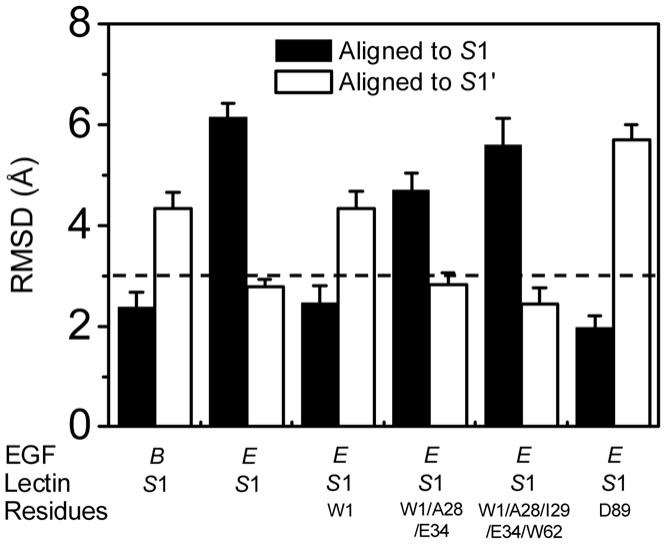
Conformational consistency of allosteric *S*1' Lec domain. Allostery was investigated by combining the equilibrated structure of *S*1 Lec domain with *E*-EGF orientation and the key residues tuned to those of *S*2 conformation. Here averaged RMSD of *R*3 heavy atom relative to crystallized *S*1 (*solid bars*) or equilibrated *S*1' from *S*1 Lec domain alone (*open bar*s) was calculated by aligning rigid regions of Lec domain for last 2-*ns* equilibration. First 5-*ns* and completed 10-*ns* simulations of *S*1 Lec domain with original (*1^st^ set of bars*) and interchanged (*2^nd^ set of bars*) EGF orientation were used as controls, respectively. A threshold of 3.0 Å (*dash line*) was plotted against *S*1' conformation to define the conformational consistency of Lec domain allostery.

Moreover, the equilibrated structures from either *S*1 Lec domain alone or “*E*-*S*1” with extended EGF domain exhibited the dramatic change of Lec domain conformation, as seen in the RMSD and displacement profiles (Figs. 2*A*–*B* and 4*B*), which proposed the existence of a potential novel structure. To test this, the equilibrated structures were compared by superposing the equilibrated Lec domain from *S*1 alone (Fig. 5*A*, *cyan*) or “*E*-*S*1” (Fig. 5*B*, *pink*) with *S*1 (*blue*) and *S*2 (*silver*) *references* (Figs. 5*A*–*B*). The sharp difference in *R*3 loop demonstrated that the equilibrated structures were distinct from the *references*. Interestingly, the conformation of equilibrated Lec domain from “*E*-*S*1” structure (*pink*) was superposed well with that from *S*1 Lec domain alone (*cyan*) (Fig. 5*C*) and the resulted RMSD of *R*3 heavy atoms was ∼2.8 Å (*2^nd^ open bar from the left* in Fig. 6). This novel conformation, denoted as *S*1' Lec domain, was characterized by the *R*3 re-orientation *via* its apex around R85 followed by the counter-clockwise rotation and the slight deviation of *R*2 apex around N57 from *S*1 (Fig. 5*A–B*). Presence of *S*1' conformation in the two systems with sufficient long simulation time (>10 *ns*) imparted the confidence that this novel conformation is a stable structure. By contrast, the equilibrated conformation from “*B-S*2” with bent EGF domain was similar to that of *S*2 Lec domain except of loosing of *R*3 loop (*data not shown*). Thus, re-orientating from *B*- to *E*-EGF domain induced the conformational changes of *S*1 Lec domain on *R*3 and *N*-terminal of *R*2 but the re-orientating from *E*- to *B*-EGF just relaxed *S*2 Lec domain at the *R*3 and the *C*-terminal of *R*2.

### Allosteric pathway of Lec domain

The emergence of *S*1' conformation from *S*1 induced by the deletion of EGF domain or re-orientation from *B*- to *E*-EGF domain proposed the possible allostery between *S*1 and *S*1'. To further test the universality of *S*1' conformation and to elucidate the allosteric pathway from *S*1 to *S*1' Lec domain, *Set IV* simulations were performed, combined with the interchanged EGF orientations, by tuning those key residues of the entrance residues W1, A28, I29, and E34 of Lec domain, the pivot residue W62 of *R*2, and the ending residue D89 of *R*3, which seemed to be crucial in the interconversion between *B*-*S*1 and *E*-*S*2 (7). One series of simulations were done for “*E-S*1” structure combined with key residues tuned to those of *S*2 conformation (Fig. 6). Averaged RMSD of *R*3 heavy atoms to crystallized *S*1 (*solid bars*) or *S*1' conformation from equilibrated *S*1 Lec domain (*open bars*) was calculated from last 2 *ns* equilibration and the threshold of 3.0 Å was used to determine if the conformational change takes place or not. It was found that, as compared to the equilibrated *S*1 conformation from *B*-*S*1 or reconstructed “*E*-*S*1” structure (*1^st^ or 2^nd^ set of bars from the left*), the additional adjustment of residue W1 or D89 orientation tuned to that of *S*2 did not induce conformational change of *S*1 Lec domain (RMSD <3.0 Å, *3^rd^* and *6^th^ solid bars*). The combined tuning of W1/A28/E34 or W1/A28/E34/W62, however, resulted in the conformational change (RMSD >3.0 Å, *4^th^* and *5^th^ solid bars*), resulting in the conformations similar with that of equilibrated *S*1 Lec domain (RMSD 3.0 Å; *open bars*) (Fig. 6). All the resulted structures were distinct from that of *S*2 Lec domain (RMSD >3.0 Å, *open bars* in Fig. S1 of File S1.). In addition to the *R*3 RMSD calculated from aligning rigid regions of Lec domain and used to determine both the conformational change and the re-orientation of *R*3, we defined an alternative RMSD by aligning *R*3 itself. The calculations supported the above observation of the transition from *S*1 to *S*1' (Fig. S2 in File S1.). Thus, our simulations suggested that no allostery takes place from *S*1 to *S*2 conformation regardless of interchanging EGF domain and/or tuning key residues. Another series of simulations for “*B-S*2” conformation were also conducted by oppositely tuning same residues of *S*2 conformation to those of *S*1 conformation. All the *R*3 RMSD was found to yield >3.0 Å when aligning the rigid regions to those of crystallized *S*1 (*solid bars*) or *S*2 (*open bars*) conformation (Fig. S3 in File S1.). Combined with the RMSD of *R*3 itself (Fig. S4 in File S1.), no allostery but only the loosing of *R*3 was observed in *S*2 Lec domain. Taken together, these results suggested that the interaction network attributed to W1 or D89 residue may not be prerequisite for allostery of Lec domain, as predicted previously [7]. It was further confirmed that the interchanged allostery between *S*1 and *S*2 structure did not take place directly and that *S*1' conformation could be a universal allosteric conformation of Lec domain originated from *S*1.

**Figure 7:**
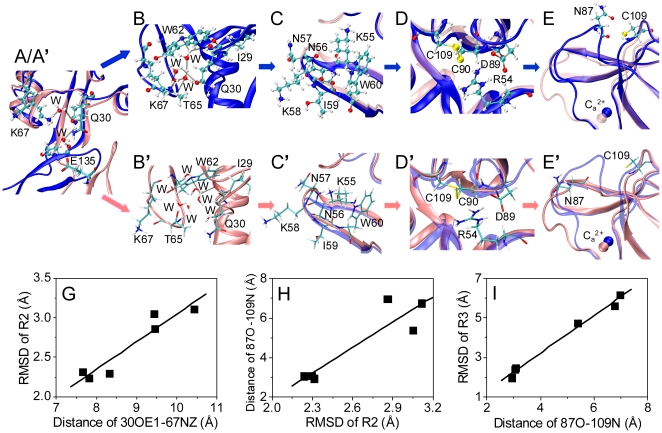
Allosteric pathway from *S*1 to *S*1' Lec domain when changing *B*- to *E*-EGF orientation. Illustrated sequentially were the *H* region between Lec and EGF domains (*A*/*A'*), the interface between *R*1 and *R*2 (*B*/*B'*), the *R*2 itself (*C*/*C'*), the interface between *R*2 and *R*3 (*D*/*D'*), and the *R*3 itself (E/E'). Conformational difference between *B*-*S*1 (*blue*) and *E*-*S*1' (*pink*) Lec domains was presented in (*A*–*E*) and (*A'*–*E'*), respectively, from final snapshots of the first 5-*ns* equilibration of *B*-*S*1 and the completed 10-*ns* equilibration simulations. Lec domain was presented as *newcartoon*, water molecules (denoted as W) and key residues as *named CPK* in (*A*–*E*) and as *named licorice* in (*A'*–*E'*), respectively, and hydrogen bonds between waters and key residues as *black dash lines*. Only Lec domains of *S*1 and *S*1', superposed into same panel for comparison, were demonstrated in *A*/*A'* and *C/C'–E/E'* for clarity. Also illustrated for the specific regions involved in allostery pathway were the correlations between distance of 30OE1-67NZ and RMSD of part of *R*2 (K55-W60) (*G*), between RMSD of *R*2 and distance of 87O-109N (*H*), and between distance of 87O-109N and RMSD of *R*3. Data (*points* in *G*–*I*) were calculated from six simulations as shown in Figure 6 and were averaged for last 2-*ns* of each simulation. RMSD for part of *R*2 or of *R*3 were calculated by aligning the rigid regions of simulated Lec domain to those of crystallized *S*1 Lec domain.

Then, what's the underlying allosteric pathway from *S*1 to *S*1' conformation? Further analyses demonstrated that the allostery was initiated by the breakage of water bridge among Q30 of *R*1, K67 of *R*2, and E135 near *H* region and resulted in extending EGF domain (*pink newcartoon* and *CPK residues* in Fig. 7*A* *'*). Originally the water bridge networks between Q30 and K67 and between Q30 and E135 in *B*-*S*1 structure (*blue newcartoon* and *licorice residues* in Fig. 7*A*) formed a closed cavity with several water molecules tightly linking *R*1 and part of *R*2 (W62-K67) (Fig. 7*B*). The disruption of water bridge near *H* region induced the opening of cavity gate by separating side-chains of K67 and Q30 and swallowed additional water molecules (Fig. 7*B* *'*). It also weakened the interaction between *R*1 and *R*2 loop (W62-K67) and pushed rotationally *R*2 away from original location (Figs. 7*C and 7C* *'*). The motion of *R*2 was then transmitted to the loop (H108-K113) through the β_7_ and β_8_ sheets *via* strong Hbond interaction between R54 of *R*2 and D89 of *R*3 and the disulfated bond between C90 and C109, which, in turn, pushed C109 residue away (Figs. 7*D and 7D* *'*) and disrupted the interaction of C109 backbone Hbond with N87 of *R*3. The release of N87 from C109 resulted in the *R*3 re-orientation and, finally, terminated the allostery of Lec domain (Figs. 7*E and 7E* *'*). In short, the destroy of hinge water bridge induced the opening of cavity gate governed by Q30-K67 interaction and weakened the interaction between *R*1 and part of *R*2 (W62-K67), followed by the re-orientation of *R*2 transferred to C109 *via* the interaction of *R*2 N-terminal and *R*3 C-terminal as well as the rigid C90-C109 disulfated bond, which finally activated the conformational change of *R*3 through the breakage of N87-C109 Hbond. Such an allosteric pathway was further confirmed by the following correlations: The re-orientation of *R*2 (defined by RMSD of part of *R*2 (K55-W60)) was reinforced with width of cavity gate (defined by distance between two atoms of Q30OE1 and K67NZ) (Fig. 7*G*); The breakage of N87-C109 Hbond (denoted as distance between two atoms of N87O and C109N) was enforced by *R*2 re-orientation (Fig. 7*H*); And the allostery of *R*3 (defined by RMSD of *R*3) correlated positively with N87-C109 Hbond disruption (Fig. 7*I*).

### Spontaneity and universality of allostery from *S*1 to *S*1'

**Figure 8:**
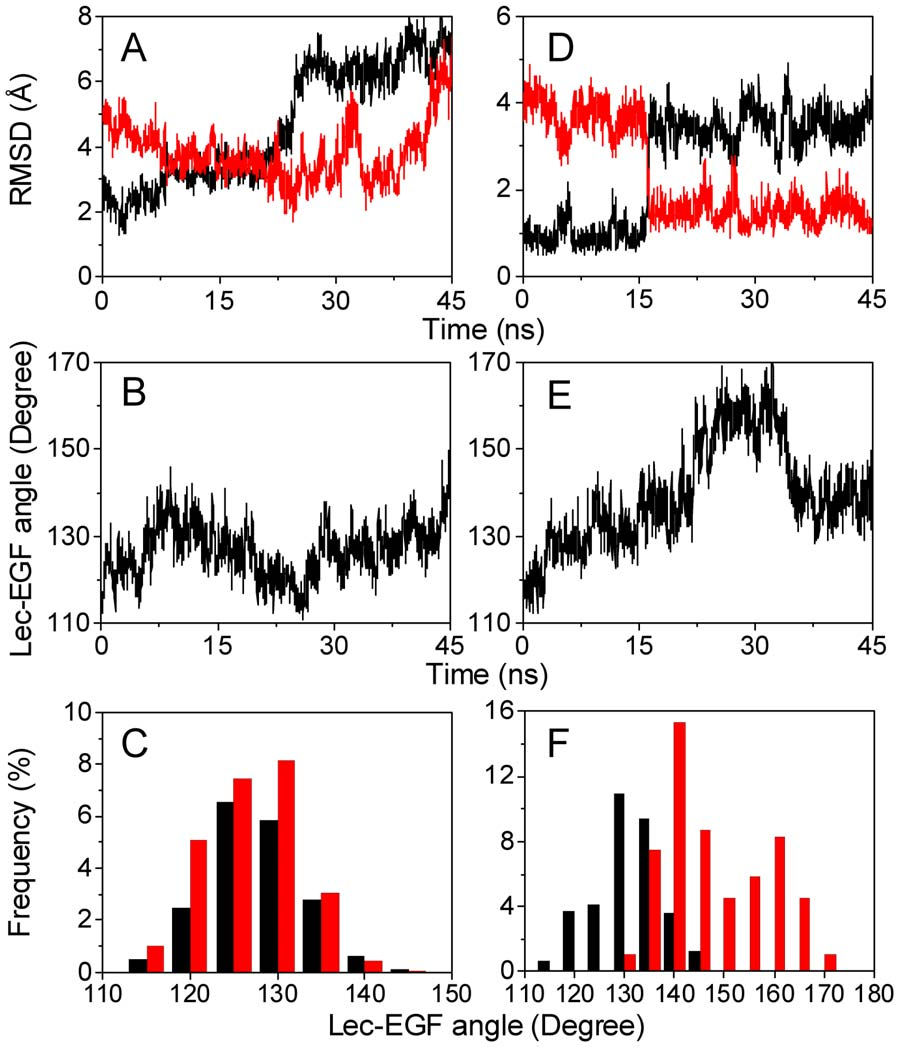
Allosteric spontaneity and universality from *S*1 to *S*1' Lec domain for P- (*A*–*C*) and E-selectin (*D*–*F*). RMSD of *R*3 heavy atoms relative to crystallized *S*1 (*black*) or equilibrated *S*1' (*red*) was calculated by aligning rigid regions of Lec domain (*A*, *D*). EGF orientation was calculated as the angle between Lec and EGF domain (*B*, *E*). Distribution of EGF orientation was plotted as the frequency of Lec-EGF angle in a bin size of 5° corresponding to Lec conformation assigned to *S*1 (*black*) or to S1' (*red*) (*C*, *F*). A single snapshot was assigned to *S*1 conformation when the *R*3 RMSD yielded ≤3.5 or 2.5 Å to *reference S*1 and ≥4.0 or 3.0 Å to *S*1', or assigned to *S*1' conformation when *vice versa*, for P- (*C*) and E-selectin (*F*), respectively.

Simulations of *S*1 Lec domain by deleting EGF domain (Fig. 2*A, B*) and reconstructed “*E*-*S*1” structure by reorienting EGF domain from bent to extended conformation combined with tuning the key residues (Figs. 4*A*–*B*, 6, and S1–S2 in File S1.) demonstrated the stability and university of *S*1' Lec conformation. It is still possible, however, that the novel conformation was imposed seemingly by manually deleting the EGF domain or modulating its orientation. To exclude the possibility, additional simulations were performed on crystallized *B*-S1 and *E*-*S*2 structures without any constraints (*Set V* in Table 1). Similar to those for *S*2 Lec domain (Figs. 2*C*–*D*) and reconstructed “*B*-*S*2” structure by reorienting EGF domain from extended to bent conformation combined with tuning key residues (Figs. S3–S4 in File S1.), 30 *ns* equilibration of *E*-*S*2 exhibited similar relaxation of *R*3 loop without intrinsic conformational change (*1^st^ sets of bars* in Figs. S3–S4 in File S1.). 45 *ns* equilibration of *B*-*S*1, however, demonstrated the spontaneous change of Lec domain conformation as well as the reorientation of EGF domain after ∼5 *ns* fluctuation around *B*-*S*1 structure (Figs. 8*A*–*B*). Specifically, the Lec domain started to deviate from *S*1 for ∼15 *ns* transition (7.5–20.5 *ns*) and then evolved into *S*1' around 25 *ns*, with the RMSD of *R*3 heavy atoms to *S*1 (*black*) and *S*1' (*red*) running up to ∼6.2 *Å* and down to ∼2.8 *Å*, respectively (Fig. 8*A*). In contrast to the *S*1' conformations resulted from EGF deletion or reorientation, the *S*1' conformation spontaneously obtained from *B*-*S*1 equilibration was unstable sufficiently, which finally evolved away into other conformations with high RMSD of *R*3 fluctuating to ∼5.8 Å around 31 *ns* and even higher value during last 3 *ns* (Fig. 8*A*, *red*). EGF orientation also exhibited the multiple phases with more than three times of transition between bent (∼122°) and extended (∼138°) conformations (Fig. 8*B*). Thus, the novel *S*1' conformation could exist spontaneously with less stability.

Crystallized Lec-EGF domains of E-selectin liganded with sLe^X^ presented the similar characteristics with *B*-*S*1 of P-selectin in both Lec conformation and EGF orientation (6). Here a 45 *ns* simulation for E-LE domains was performed to further test the universality of *S*1' allostery from *S*1 in different selectin members. The RMSD of *R*3 heavy atoms relative to P-selectin *S*1 (*black*) and *S*1' (*red*) conformation indicated that E-selectin Lec domain also experienced the spontaneous allostery to *S*1' after ∼15 *ns* fluctuation around *S*1, where the allostery happened sharply and the *S*1 and *S*1' conformations were ultimately distinct (Fig. 8*D*). E-selectin EGF domain re-oriented to the high angle from the initial 120° and presented more extended angles with the maximum of ∼165° at ∼30 *ns* than that of P-selectin (Fig. 8*E*).

We further correlated the allostery of Lec domain to *S*1' with the reorientation of EGF domain to extended states, as observed in the simulations of P- and E-LE domains. The distribution of EGF orientation of *S*1' conformation (*red*) overlapped partially to that of *S*1 conformation (*black*) in both P- (Fig. 8*C*) and E- LE domains (Fig. 8*F*), suggesting that not all the large (or small) Lec-EGF angles are necessarily corresponding to *S*1' (or *S*1) conformation. It was also found that the distribution shifted rightward from *S*1 to *S*1' conformation, implying that *S*1' conformation favors more extended EGF orientation and that the extension of EGF orientation promotes the possibility of allostery from *S*1 to *S*1'. Moreover, the slight (Fig. 8*C*) or dramatic (Fig. 8*F*) shifting in the distribution was well correlated with the transient (Fig. 8*A*) or sharp (Fig. 8*D*) difference in the RMSD for P- or E-LE domains. Taken together, our simulations indicated that allostery from *S*1 to *S*1' could happen spontaneously, which is universal for both P- and E-selectin.

## Discussion

Allostery is essential for a protein to present different functional states. For example, catch bond behavior (bond lifetime increases with applied force) of bacterial FimH was attributed to the conformational change of Lec domain induced by pilin domain [8], [19]. Allostery of interdomain region of vWF A1 domain possibly regulated catch bond behavior with platelet glycoprotein Ib (GPIbα) [19], [20]. Activation of headpiece of integrin α_5_β_1_ contributed to the presence of catch bond with fibronectin ligand [21]. Specifically, catch bond nature of a selectin molecule, first visualized in the forced dissociation of P- [22] or L-selectin [23] from PSGL-1, was correlated biologically with a shear-threshold feature for leukocyte tethering and rolling adhesion mediated by selectin-ligand interactions [24], [25]. An allosteric model for catch bond of P-selectin-PSGL-1 interaction was proposed from analyzing the two crystallized P-LE structures, which assumed that the high-affinity conformation of P-LE (*S*2) induced by forced allostery from its low-affinity conformation (*S*1) is favored to its ligation [7]. While a few measurements confirmed the occurrence of P-selectin allostery [13], [14], it is still unknown if the interchanged transition between *S*1 and *S*2 takes place directly along the predicted pathway and what the dynamic pictures are. Thus, MD simulations of P- and E-LE were performed in the current study, attempting to visualize the dynamic pathways of structural allostery for Lec domain. By elucidating the impacts of the stability of Lec domain alone, the ligation of SGP-3 ligand, the orientation of EGF domain, and the stability of LE domains, our results indicated that the allostery of Lec domain existed and the orientation of EGF domain induced the conformational change of Lec domain. Three novel observations were found in the simulations: the first is that the interchanged transition between *S*1 and *S*2 was unable to take place directly but a stable, novel conformation (*S*1') was presented originating from *S*1, the next is that the conformation of equilibrated *S*1 Lec domain alone was similar with that of equilibrated *S*1' conformation when EGF domain for *S*1 was tuned from *B* to *E* orientation (Figs. 4 and 5), and the final is that the allostery from *S*1 to *S*1' and the reorientation of EGF domain could happen spontaneously where the *S*1' conformation favors the extended EGF orientation (Fig. 8).

At least three lines of evidence were proposed from experimental measurements for the co-existence of multiple conformations of P-Lec EGF domains. First, both the slow and fast phases concurrently exist in the dissociation kinetics for a wild-type P-selectin construct (in the order-of-magnitude of ∼10^−1^ and ∼10^−2^ s^−1^) or a wedge mutant that opens the interface between Lec and EGF domains (in the magnitude of ∼10^−2^ and ∼10^−3^ s^−1^) [13], suggesting that at least three kinetic phases exist to correspond to three stable conformations of the protein containing Lec, EGF and SCR1 domains. Similar measurements were also obtained for a cleft P-selectin mutant with Lec, EGF, and SCR1-2 domains that opens within Lec domain [14] where the kinetic model used to probe the two equilibrated conformations (bent and extended) for binding to and dissociating from the chip surface is no longer applicable to estimate the reliable parameters, indicating that more than two P-selecitn conformations may exist. Second, not only the existence of EGF domain is pre-requisite for sufficient binding of P-selectin to its liagnd [2], [3], but the substitution of EGF domain of L-selectin with the homologous domain from P-selectin also enhances the binding to L-selectin ligand under shear flow while its equilibrium features toward soluble ligands remains the same [26], implying that the cooperativity of Lec and EGF domains promotes different kinetic phases. Third, a recent report on a triphasic force dependence of lifetime of E-selectin-ligand bond (personal communications) further supported that multiple stable conformations of P-selectin be expected from the experiments. Several testable predictions such as crystallizing the *S*1' structure or characterizing the high-affinity phase for P-LE wedge cleft can be proposed to elucidate the biological significance of the novel *S*1' conformation in future studies.

Dynamic allosteric pathways found in the current study were also different from those proposed by comparing the conformational differences of crystallized *B-S*1 and *E-S*2 structures [7]. In the previous prediction, the extending from *B-* to *E-*EGF orientation induced directly the allostery of Lec domain from *S*1 to *S*2 *via* sequential interactions along the following pathway: First, the extending orientation broke up the interaction network near *H* region between EGF and Lec domains, followed by the disruption of interaction among residues W1, A28, and E34 *via* a water molecule. Next, the resulted re-orientation of *R*1 loop drove the tuning of W62 side-chain and then pushed the anti-parallel β-sheets of *R*2 away to re-distribute of hydrogen network of R54-D89 and K55-N83 between *R*2 and *R*3. Finally, the re-orientation of *R*3 induced the conformational transition from *S*1 to *S*2. Our MD simulations, however, proposed a distinct allosteric pathway. Here the side-chain re-orientation of residues W1 and R89 (the two key residues assumed to be responsible for the allostery [7]) did not induce the conformational change of Lec domain from *S*1 to *S*2 or *S*1', indicating that the breakage of either the interaction network of W1 around *H* region or the linkage between *R*2 and *R*3 *via* R89 was not required for allostery. By contrast, re-orientation of residue W62 (the pivot residue involved in the allostery [7]) promoted the conformational change from *S*1 to *S*1', implying that this residue was engaged in the allosteric pathways *via* pushing *R*2 away sufficiently. This alternative pathway was consistent with the previous observations that both the A28H mutation [14] and the introduction of a glycan wedge between EGF and Lec domains [13] resulted in the allostery of P-selectin by destroying the closed cavity between *R*1 and *R*2 loops (W62-K67). Conclusively, the five sets of simulations (∼275 *ns* totally) defined well the specificity and stability of *S*1' conformation and the allosteric analyses indicated that the novel *S*1' conformation takes place along a different pathway.

While the allostery of Lec domain from *S*1 to *S*1' could happen universally, is it also possible to further induce the conformational change from *S*1' to *S*2? PSGL-1 ligation is one of potential pathways since the ligation of Lec domain makes it possible to alter the conformation of Lec domain. For example, the fluorescent intensity of labeled P-selectin was enhanced up to 10–13% when binding to PSGL-1 ligand [27], implying that the conformational change might occur within P-selectin molecule. Regardless of that *S*2 conformation was stabilized by the presence of SGP-3 ligand (Fig. 3), however, our results seemed not to support the conformational transition from *S*1 or *S*1' to *S*2 in the equilibration simulations of *S*1, *B*-*S*1, “*E*-*S*1”, or “*E*-*S*1'” when ligated to SGP-3 (Figs. 3, and S5 in File S1.). Another possibility lie in the exertion of applied force that may be required to induce the allostery to *S*2 in the forced dissociation of Lec-SGP-3 complexes [7]. Again, the expected transition to *S*2 did not take place in our SMD simulations of *B*-*S*1-SGP-3 or *E*-*S*1'-SGP-3 complex (Fig. S6 in File S1.). Instead, the *S*1 conformation could evolve into *S*2-like *via S*1' conformation in the forced unfolding of EGF domain probably due to the resulted large Lec-EGF angle (*cf*. supplementary text and Fig. S7 in File S1.). In fact, the prolonged equilibration simulation of *B*-*S*1 exhibited the similar transition at the end phase of simulation (∼43–45 *ns*) (Fig. 8*A*–*B*). Noting that the allostery from *S*1' to *S*2-like conformation followed the extension of EGF orientation, it is reasonable to assume that the allostery from *S*1 to *S*1' and further to *S*2-like conformation could happen spontaneously and the three conformations favor different EGF orientations where the *S*2-like conformation corresponding to the most extended EGF domain.

Our findings of an alternative allostery of P-selectin Lec domain also supported the prediction that the presence of EGF domain regulated the P-selectin-PSGL-1 binding by changing the conformation of Lec domain upon EGF orientation [7], where the *S*1' conformation favored the extended EGF orientation than that of *S*1 conformation (Fig. 8). This allosteric model can be used to interpret the catch bond nature since high force promotes strong and long-lived P-selectin-PSGL-1 interaction by turning EGF domain to extended orientation and then inducing the corresponding allostery of Lec domain. Besides, our simulations were not inconsistent with another sliding-rebinding model of catch bond where the forced opening of interdomain *H* hinge promoted the formation of new interactions to slow down unbinding and to prolong bond lifetime [28]. While the sliding-rebinding model and the corresponding measurements [29] supported that the flexibility of interdomain hinge and the extension of EGF-Lec angle tilted the binding interface to the direction of external force and allowed the two contact sides sliding against each other, our simulations proposed the possibility for conformational change of Lec domain upon EGF re-orientation to alter the interaction. Future integration of the three structural or allosteric models is required to elucidate the intrinsic mechanisms of conformational changes in dominating P-selectin-PSGL-1 interaction.

Finally, the current study provided the dynamic pictures of P-selectin EGF and Lec structures to visualize the conformational change of Lec domain from the viewpoints of EGF orientation, interaction network of EGF-Lec interdomain, and interaction between P-LE and SGP-3 ligand. It was indicated that *S*1 and *S*2 structures are unable to interchange directly but able to go through a novel conformation of *S*1' Lec domain with an alternative allosteric pathway. Our results furthered our understanding in the structure-function relationship of P-selectin.

## Supporting Information

File S1 Supplementary Text and Supplementary Figures of S1-S7.
(DOC)
