# Supplementary material for: Visualization of Allostery in P-Selectin Lectin Domain Using MD Simulations
Source: PLoS One. 2010 Dec 8;5(12):e15417. doi: 10.1371/journal.pone.0015417 (PMC2999562; doi:10.1371/journal.pone.0015417)
Supplement: File S1 — Supplementary Text and Supplementary Figures of S1-S7. (DOC) [file pone.0015417.s001.doc]

**Supplemental Text**

Noting that the extended EGF orientation favored *S*1’ conformation (Fig. 8), the SMD simulation for forced unfolding of EGF domain in *B*-*S*1 structure was performed using *cf*-SMD algorithm at 100 pN along the vector from a fixed atom of L116-C_α_ to a pulled atom of D158-C_α_ and three distinct conformations were visualized. The RMSD evolution of *R*3 heavy atoms (Fig. S7*A*) relative to *S*1 (*black*), *S*1’ (*red*), and *S*2 (*blue*), together with the snapshots (Figs. S7*C*-*E*), indicated that the Lec domain conformation exhibited the stable *S*1’ conformation at ~11.5-30 *ns* (Fig. S7*D*) through a *S*1 phase at ~0-3 *ns* (Fig. S7*C*) followed by a transition phase at 3-11.5 *ns*, and further a new conformation beyond 30 *ns* (Fig. S7*E*). EGF domain re-oriented quickly into a large angle of ~ 158º at > 10 *ns* (Fig. S7*B*). Conformational superposition illustrated that the new conformation beyond 30 *ns* tended to be the *S*2-like conformation with similar orientation of *R*3 loop toward binding pocket of Lec domain (Fig. S7*E*).

**Supplemental Figures**

**Figure S1.** *Impact of key residues on allostery of S1 Lec domain.* Conformational change of equilibrated *S*1 Lec domain with *E*-EGF orientation combined with the key residues tuned to those of *S*2 conformation. Here averaged RMSD of *R*3 heavy atom relative to crystallized *S*1 (*solid bars*) or S2 (*open bar*s) *references* was calculated by aligning rigid regions of Lec domain for last 2-*ns* equilibration. First 5-*ns* and completed 10-*ns* simulations for *S*1 Lec domain with original (*1^st^ set of bars*) and interchanged (*2^nd^ set of bars*) EGF orientation were used as controls. A threshold of 3.0 Å (*dash line*) was plotted against crystallized *S*1 conformation to define the occurrence of Lec domain allostery.

**Figure S2.** *Conformational change of allosteric S1’ Lec domain.* Conformational change of equilibrated *S*1 Lec domain with *E*-EGF orientation combined with the key residues tuned to those of *S*2 conformation. Here averaged RMSD of *R*3 heavy atom relative to crystallized *S*1 *reference* was calculated by aligning *R*3 itself for last 2-*ns* equilibration. First 5-*ns* and completed 10-*ns* simulations for *S*1 Lec domain with original (*1^st^ bar*) and interchanged (*2^nd^ bar*) EGF orientation were used as controls.

**Figure S3.** *Impact of key residues on the stability of S2 Lec domain.* Conformational change of equilibrated *S*2 Lec domain with *B*-EGF orientation combined with the key residues tuned to those of *S*1 conformation. Here averaged RMSD of *R*3 heavy atom relative to crystallized *S*1 (*solid bars*) or *S*2 (*open bar*s) *reference* was calculated by aligning rigid regions of Lec domain for last 2-*ns* equilibration. Simulations for *S*2 Lec domain with original (*1^st^ set of bars*) and interchanged (*2^nd^ set of bars*) EGF orientation were used as controls. A threshold of 3.0 Å (*dash line*) was plotted against crystallized *S*2 conformation to define the conformational change of Lec domain.

**Figure S4.** *Conformation of S2’ Lec domain.* Conformational change of equilibrated *S*2 Lec domain with *B*-EGF orientation combined with the key residues tuned to those of *S*1 conformation. Here averaged RMSD of *R*3 heavy atom relative to crystallized *S*2 *reference* was calculated by aligning *R*3 itself for last 2-*ns* equilibration. Simulations for *S*2 Lec domain with original (*1^st^ bar*) and interchanged (*2^nd^ bar*) EGF orientation were used as controls.

**Figure S5.** *Impact of SGP-3 binding on allostery of Lec domain from S1 or S1’ to S2.* Conformational change of equilibrated structures of SGP-3-liganded *S*1 Lec domain alone (*A*), *S*1 Lec domain with original *B*-EGF (*B*) and interchanged *E*-EGF (*C*) orientation, and *S*1’ Lec domain with *E*-EGF orientation (*D*). Here RMSD of *R*3 heavy atom to *S*2 was calculated by aligning rigid regions of Lec domain.

**Figure S6.** *Impact of forced SGP-3 dissociation on Lec domain allostery from S1 or S1’ to S2.* Conformational change of *S*1 Lec domain with original *B*-EGF (*A*) or *S*1’ Lec domain with *E*-EGF orientation (*B*) dissociating from SGP-3 ligand. Here RMSD of *R*3 heavy atom to *S*2 was calculated by aligning rigid regions of Lec domain. SMD simulations of dissociation of P-LE-SGP-3 complex were performed using *cv*-SMD algorithm by pulling C-terminal atom P618-C_α_ of SGP-3 ligand peptide *via* a spring with a spring constant of 70 pN/Å at a constant speed of 0.01 Å/*ps* along the vector from fixed point of *C*-terminal atom D158-C_α_ of EGF domain to the pulled end.

**Figure S7.** *Impact of forced EGF unfolding on Lec domain allostery from S1 to S1’ or S2.* SMD simulations of forced unfolding of EGF domain of *B*-*S*1 structure. RMSD of *R*3 heavy atoms to *S*1 (*black*), *S*1’ (*red*), and *S*2 (*blue*) were calculated by aligning rigid regions of Lec domain to evaluate the conformational change of *S*1 Lec domain (*A*). EGF orientation was identified by calculating the Lec-EGF angle as illustrated in Figure 1*A* (*B*). Conformational evolution of Lec domain was illustrated by superposing the snapshots (*pink*) at 2 (*C*), 25 (*D*) and 36 ns (*E*) (corresponding to *arrows* in *A*) with crystallized *S*1 (*blue*), *S*2 (*silver*), and equilibrated *S*1’ (*cyan*). *R*3 was presented as *thick, opaque* *newcartoon* and the others were illustrated as *transparent* *newcartoon* for clarity. Simulation of unfolding of *B*-*S*1 was performed using *cf*-SMD algorithm at a constant force of 100 pN.
